# Discovery and SARs of *Trans*-3-Aryl Acrylic Acids and Their Analogs as Novel Anti- *Tobacco Mosaic Virus* (TMV) Agents

**DOI:** 10.1371/journal.pone.0056475

**Published:** 2013-02-13

**Authors:** Meng Wu, Ziwen Wang, Chuisong Meng, Kailiang Wang, Yanna Hu, Lizhong Wang, Qingmin Wang

**Affiliations:** State Key Laboratory of Elemento-Organic Chemistry, Research Institute of Elemento-Organic Chemistry, Nankai University, Tianjin, China; Institut Pasteur, France

## Abstract

A series of *trans*-3-aryl acrylic acids **1**–**27** and their derivatives **28**–**34** were prepared and evaluated for their antiviral activity against *tobacco mosaic virus* (TMV) for the first time. The bioassay results showed that most of these compounds exhibited good antiviral activity against TMV, of which compounds **1**, **5**, **6**, **20**, **27** and **34** exhibited significantly higher activity against TMV than commercial Ribavirin both *in vitro* and *in vivo*. Furthermore, these compounds have more simple structure than commercial Ribavirin, and can be synthesized more efficiently. These new findings demonstrate that *trans*-3-aryl acrylic acids and their derivatives represent a new template for antiviral studies and could be considered for novel therapy against plant virus infection.

## Introduction

**Figure 1:**
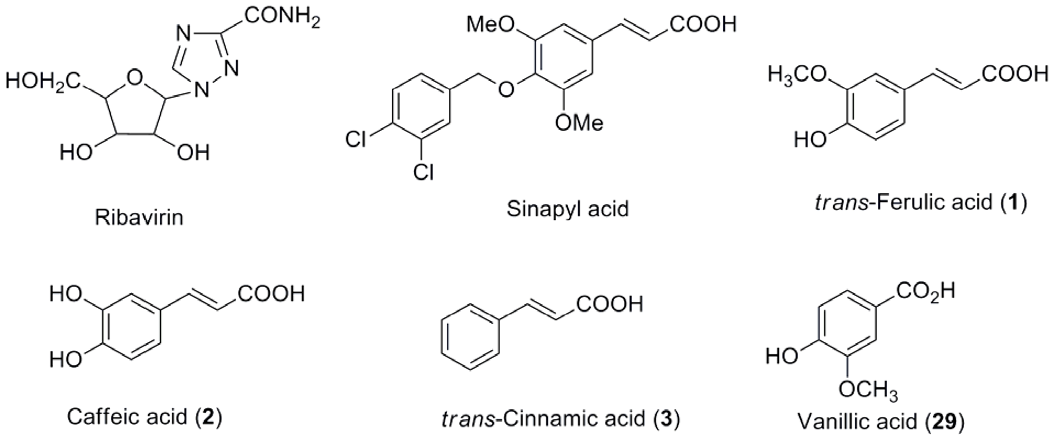
Chemical structure of Ribavirin, acrylic acids 1–3 and acid 29.

Plant viruses cause dramatic losses in agriculture and horticulture all over the world [1]. *Tobacco mosaic virus* (TMV), one of the most well-studied plant viruses [2], infects more than 400 plant species belonging to 36 families, such as tobacco, tomato, potato, and cucumber [3], [4]. As a successfully registered plantviral inhibitor, Ribavirin (Figure 1) is widely used to prevent TMV disease [5]. However, the inhibitory effects of Ribavirin are less than 50% at 500 µg/mL, and its effective duration is not long. In fact, there are no super chemical treatments that can absolutely inhibit TMV once it has infected plants. Therefore, the development of highly efficient, novel, environmentally benign antiviral inhibitors has been continuously conducted. During the process for finding an effective way to protect plants from TMV infection, Song et al. have also reported that cyanoacrylate derivatives and amide derivatives containing α-aminophosphonate moiety exhibited moderate to excellent antiviral activity against TMV [6], [7]. Natural phenanthroindolizidine alkaloids have been proved to be efficiency to inhibit TMV by our group [8], [9].

Natural product-based agrochemicals offer advantages in that they can sometimes be specific to a target species and often have unique modes of action with little mammalian toxicity. Another benefit is their ability to decompose rapidly, thereby reducing their risk to the environment [10], [11].

*trans-*3-(Substitutedphenyl)acrylic acids and their derivatives are widely distributed in the plant kingdom and are found to have diverse biological activities. These natural or natural-based compounds have been applied widely in medicinal formulation for their properties of low toxicity and environmental friendly [12]. For example, *trans-*ferulic acid (**1**, Figure 1) ubiquitously distributed in primary plant cell walls and crop bran [13] exhibited diverse physiological activities such as reduction of serum cholesterol levels [14], antioxidant properties in several oil models [15], antibacterial [16] and anticancer activity [17], [18]. *trans-*Cinnamic acid (**3**, Figure 1) also was found to have allelopathy activity [19], moderate to strong insecticidal activity [20], and a significant inhibitory effect on phenylalanine ammonia-lyase activity in wheat seedlings [21]. Sinapyl acid (Figure 1) possessing the same α, β-unsaturated carbonyl as an important Michael acceptor anticancer pharmacophore displayed potent cytotoxicity against the KB cell line with an IC_50_ value of 14 µM [22]. Vivanco reported that *trans*-cinnamic acid, *o*-coumaric acid and ferulic acid exhibited antimicrobial activity against both soil-borne bacteria and fungi [23]. Antitumor activities of various *trans-*3-(substitutedphenyl)acrylic acids derivatives were also explored by many research groups [24]–[28]. Hydroxycinnamic acid esters are widely distributed in the plant kingdom and are reported as cellular antioxidants, anti-inflammatory agents, or inhibitors of enzymes involved in cell proliferation [29]–[31]. However, up to now, no one has reported the antiplantviral activity of the *trans-*3-(substitutedphenyl)acrylic acids or their derivatives.

During our research for potent antiplantviral remedies, we found that *trans-*ferulic acid showed excellent antiviral activity against TMV. Based on this finding, a series of *trans*-3-aryl acrylic acids **1**–**27** and their derivatives **28**–**34** were designed, synthesized and systematically evaluated for their antiviral activity against TMV.

## Results and Discussion

### Chemistry

**Figure 2:**
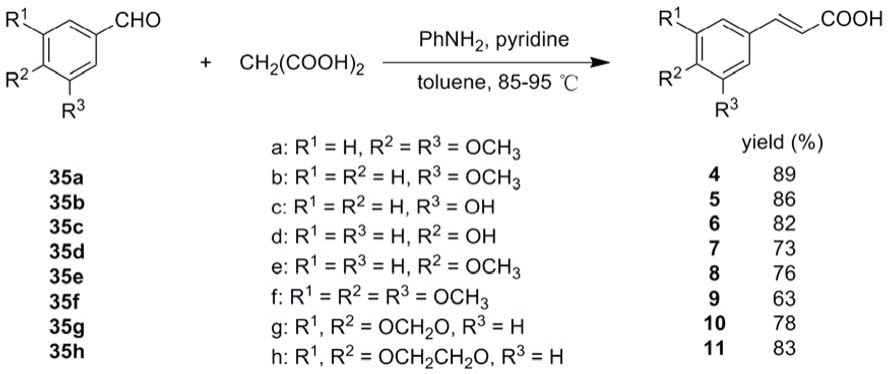
Synthesis of trans-3-(substitutedphenyl)acrylic acids (4–11).

**Figure 3:**
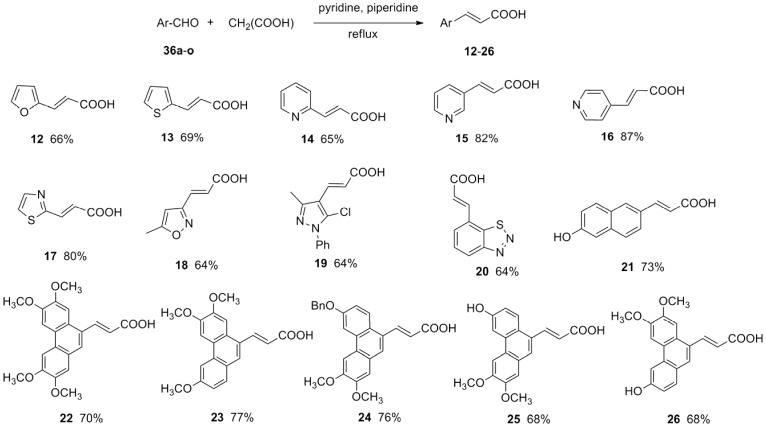
Synthesis of *trans*-3-aryl acrylic acids (12–26).

**Figure 4:**
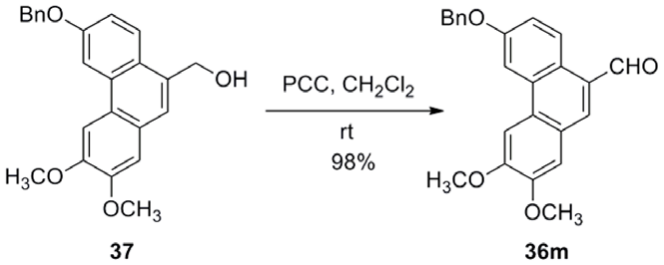
Synthesis of 6-benzyloxy-2,3-dimethoxyphenanthren-9-carboxaldehyde (36m).

**Figure 5:**
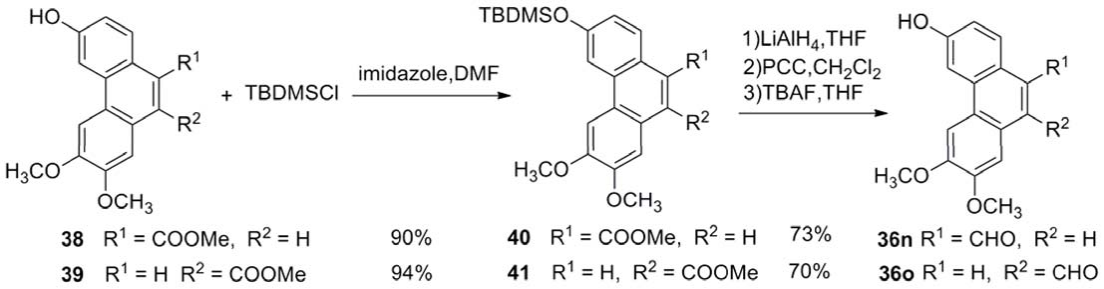
Synthesis of 2,3-dimethoxy6-hydroxyl-phenanthren-9-carboxaldehyde (36n) and 6,7-dimethoxy3-hydroxyl-phenanthren-9-carboxaldehyde (36o).

(For experiment details please see Supporting Information: Text S1) *trans*-Ferulic acid (**1**), caffeic acid (**2**), *trans*-cinnamic acid (**3**) and vanillic acid (**29**) are commercially available and other *trans-*3-aryl acrylic acids were synthesized via Knoevenagel reaction (Figure 2 and Figure 3). The aromatic aldehydes **35a**–**h**, **36a**–**h**, **36p** and **36j** are commercially available. 1,2,3-Benzothiadiazole-7-carboxaldehyde (**36i**) [32], 2,3,6,7-tetramethoxy-9-phenanthrenecarboxaldehyde (**36k**) [33] and 3,6,7-trimethoxy-9-phenanthrenecarboxaldehyde (**36l**) [33] were synthesized according to reported literatures. (6-Benzyloxy-2,3-dimethoxyphenanthren-9-yl)methanol (**37**), 6-hydroxy-2,3-dimethoxyphenanthrene-9-carboxylic acid methyl ester (**38**) and 3-hydroxy-6,7-dimethoxyphenanthrene-9-carboxylic acid methyl ester (**39**) were prepared according to our reported literature [34]. The synthetic procedure of phenanthrenecarboxaldehyde **36m**–**o** is shown in Figure 4 and Figure 5.

**Figure 6:**
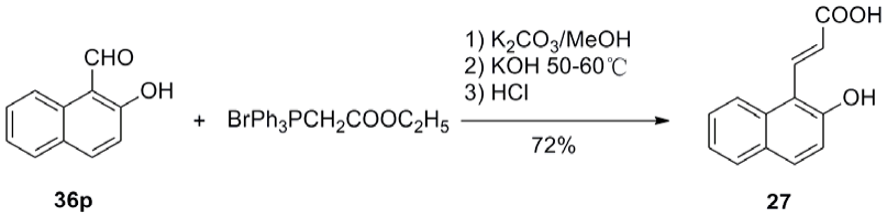
Synthesis of *trans*-3-(2-hydroxyl-1-naphthyl)acrylic acid (27).

*trans*-3-Aryl acrylic acids **4**–**26** were obtained employing aromatic aldehydes **35a**–**h**, **36a**–**o** and malonic acid as starting materials via classical Knoevenagel condensation (Figure 2, Figure 3). However, *trans*-3-(2-hydroxyl-1-naphthyl)acrylic acid (**27**) could not be obtained in good yield via Knoevenagel condensation. Then wittig reaction was applied and **27** was prepared from aldehyde **36p** in good yield (Figure 6). Most of the aromatic aldehydes were commercially available or prepared according to published procedure, only the preparation of phenanthryl aldehydes **36m**–**o** was described here. **36m** was prepared through a PCC oxidation from corresponding alcohol **37** [28] which was an intermediate of phenanthroindolizidine alkaloids (Figure 4). **36n** and **36o** were prepared from corresponding carboxylates **38** and **39** by the conventional four steps (protection, reduction to alcohol, oxidation to aldehyde and deprotection) in about 65% overall yield (Figure 5).

**Figure 7:**
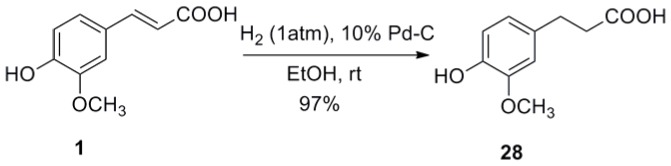
Synthesis of compound 28.

**Figure 8:**
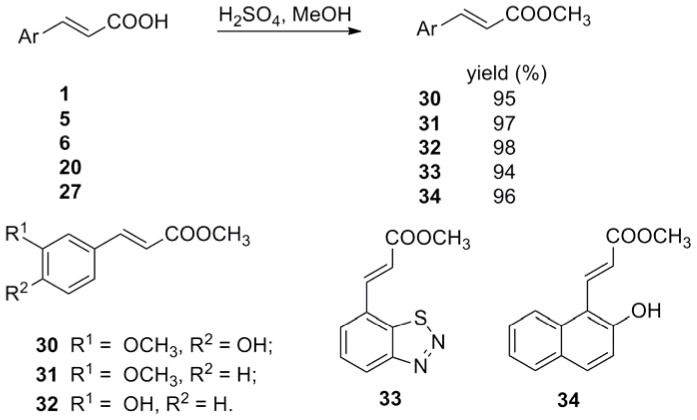
Synthesis of *trans-*3-aryl methylacrylate 30–34.

Substituted benzaldehydes **35a**–**h** were used to get *trans-*3-substituted phenyl acrylic acids **4**–**11**. To further investigate the effect of benzene ring on antiviral activity, a series of simple aromatic rings such as furan ring, thiophene ring, pyridine ring, thiazole ring, isoxazole ring, pyrazole ring, benzothiadiazole ring and naphthalene ring were chose to form *trans*-3-aryl acrylic acids **12**–**21**. As the phenanthroindolizidine alkaloids and their derivatives were reported to have excellent antiviral activity against TMV by us [8], [9], the phenanthrene rings with different substitutes were also chose to form *trans*-3-aryl acrylic acids **22**–**26**. In order to investigate the importance of the acrylic acid fragment, the carbon-carbon double bond of *trans*-ferulic acid (**1**) was hydrogenated to form **28** (Figure 7) and the *trans*-3-aryl acrylic acids **1**, **5**, **6**, **20** and **27** was esterified to get the corresponding *trans-*3-aryl acrylic acid methyl esters **30**–**34** (Figure 8).

### Phytotoxic Activity

All the *trans*-3-aryl acrylic acids **1**–**27** and their derivatives **28**–**34** were tested for their phytotoxic activity against *Nicotiana tabacum var Xanthi nc*, the results indicated that these natural product-based compounds have no phytotoxic activity at 500 µg/mL.

### Antiviral Activity *In Vitro* And *In Vivo*

The *in vitro* antiviral results of all the *trans*-3-aryl acrylic acids **1**–**27** and their derivatives **28**–**34** against TMV were listed in Table 1. To make a judgment of the antiviral potency of the synthesized compounds, the commercially available plant virucide Ribavirin was used as the control. Our results indicated that 3-(3-methoxyphenyl)acrylic acid (**5**) possesses significantly higher *in vitro* antiviral activity (68.4%) against TMV than Ribavirin (38.5%) at 500 µg/mL. At the same concentration, FA (**1**) containing hydroxyl, methoxy at the 3, 4-positions of benzene ring, compound **6** containing hydroxyl at the 3-position of benzene ring, compound **20** containing benzothiadiazole ring, compound **27** containing hydroxyl at the 2-position of naphthalene ring, and compound **24** containing benzyloxyl at 3-position of phenanthrene ring also showed higher antiviral activity (47.5%, 40.5%, 52.2%, 45.8% and 42.8%, respectively) than Ribavirin. And the compounds **3**, **22**, **23**, **25**, **26** showed *in vitro* antiviral activity close to Ribavirin. Other *trans*-3-aryl acrylic acids exhibited lower *in vitro* antiviral activity against TMV than Ribavirin. All of the methyl acrylates **30**–**34** exhibited lower *in vitro* activity than their corresponding *trans*-3-aryl acrylic acids **1**, **5**, **6**, **20**, **27**.

**Table 1:** *In Vitro* and *In Vivo* Anti-TMV Activity of Compounds **1**–**34** at 500 µg/mL.

Compd.	*In vitro* inhibition rate (%)^a^	*In vivo*		
		Inactivation effect (%)^a^	Curative effect (%)^a^	Protection effect (%)^a^
**1**	**47.5**	**38.5**	**31.2**	**34.3**
**2**	16.5	20.8	3.9	12.0
**3**	33.4	48.4	3.3	10.1
**4**	28.7	45.6	12.6	22.4
**5**	**68.4**	**46.6**	**38.9**	**30.8**
**6**	**40.5**	**34.7**	**51.7**	**32.9**
**7**	29.1	15.1	24.3	8.9
**8**	20.6	10.4	10.3	11.6
**9**	26.9	27.0	10.9	14.7
**10**	20.4	23.8	23.8	8.3
**11**	25.3	14.8	15.4	6.0
**12**	26.8	20.7	22.1	28.4
**13**	30.0	22.9	21.4	19.3
**14**	27.5	23.4	16.8	20.2
**15**	0	0	0	8.3
**16**	18.5	11.1	10.3	17.2
**17**	21.3	19.2	15.2	18.6
**18**	17.5	21.9	19.3	20.4
**19**	20.0	13.7	10.2	15.2
**20**	**52.2**	**45.0**	**46.2**	**49.7**
**21**	22.3	17.1	17.5	21.4
**22**	36.7	30.1	33.3	40.2
**23**	34.4	21.0	25.3	30.7
**24**	42.8	31.3	37.9	34.4
**25**	38.3	31.6	30.0	28.8
**26**	34.4	21.0	25.3	30.7
**27**	**50.4**	**48.8**	**42.5**	**46.6**
**28**	27.2	20.8	38.7	12.5
**29**	27.1	11.4	10.8	6.1
**30**	23.8	20.0	20.6	17.3
**31**	37.5	30.0	33.3	32.8
**32**	31.7	28.9	27.2	35.4
**33**	37.6	33.3	24.6	31.8
**34**	**45.8**	**38.9**	**40.5**	**43.2**
**Ribavirin**	**38.5**	**35.9**	**32.3**	**36.4**

(a: For details please see Supporting Information: Text S1).

The *in vivo* antiviral results of *trans*-3-aryl acrylic acids **1**–**27** and their derivatives **28**–**34** against TMV were listed in Table 1, which gave the protection effect, inactivation effect, and curative effect for the different compounds of this series. Generally, compounds **1**, **5**, **6**, **22** and **24** showed the same activity level as Ribavirin at 500 µg/mL, and compounds **20** and **27** displayed much better activity than Ribavirin in all the three effects. Interestingly, compouds **3**, **4** and **5** exhibited higher antiviral activity (48.4%, 45.6% and 46.6%) in the protection effect than Ribavirin (35.9%), while **5** showed lower activitiy in the curative effect, and **3** and **4** showed much lower activity both in the inactivation effect and curative effect. Other *trans*-3-aryl acrylic acids exhibited lower *in vivo* antiviral activity against TMV than Ribavirin.

Among compounds **1**–**11**, the more active compounds are compounds **1**, **5** and **6** which containing hydroxyl or methoxyl at the 3-position of benzene ring (**1** also containing hydroxyl at the 4-position). Removal of hydroxyl or methoxyl at the 3-position of benzene ring (**3**) caused the decrease of activity. The replacement of hydroxyl or methoxyl by methylenedioxyl or ethylenedioxyl (**10** and **11**) also caused the decrease of activity. From the structures of **5**, **6**, **7** and **8**, it can be seen that the position difference of hydroxyl or methoxyl caused great changes of activity. From the structures of **2**, **6** and **4**, **9**, it can be concluded that the increase of hydroxyl or methoxyl numbers would cause the decrease of activity. Among compounds **12**–**27**, the more active compounds are compounds **20**, **22**, **24** and **27**. That means besides benzene ring, benzothiadiazole ring, naphthalene ring and phenanthrene ring are also suitable for antiviral against TMV. Comparing the activity of **14**–**16** and **21**, **27**, it can be seen that the positions of substituents have an important effect on the antiviral activity both *in vitro* and *in vivo*. It could be concluded that the numbers and kinds of substituents also affect the antiviral activity from the structures of **22**–**26**.

To study the structure-activity relationship, different types of structures and the effects of structural changes in different regions of the molecular were considered: elimination of the double bond of the side chain gave the structures of **28** and **29**, and change of the carboxy of **1**, **5**, **6**, **20** and **27** by esterifying gave the structures of **30**–**34**. Compound **28** exhibited lower *in vitro* inhibition rate, inactivation effect and protection effect and higher curative effect than compound **1**, which indicates that the different geometry of the alkenyl vs alkyl chain may give different binding properties. Vanillic acid (**29**) displayed much lower antiviral activity, which indicates that the directly connection of carboxyl group and benzene ring is bad for antiviral activity. Compound **30**–**33** containing an ester group in the side chain were less active than their corresponding acrylic acids. Though *trans*-3-(2-hydroxyl-1-naphthyl)methylacrylate (**34**) showed higher antiviral activity than Ribavirin, it also exhibited slightly lower antiviral activity than *trans*-3-(2-hydroxyl-1-naphthyl)acrylic acid (**27**).

## Conclusion

In summary, a group of *trans*-3-aryl acrylic acids **1**–**27** and their derivatives **28**–**34** were prepared and evaluated for their antiviral activity against TMV. Most of these compounds exhibited good antiviral activity against TMV and some of them showed activity close to or even higher than Ribavirin at 500 µg/mL. A systematic SAR study on these compounds indicated that the acrylic acid fragment is important for the antiviral activity and the substituents have an important effect on the antiviral activity. Among them, compounds **1**, **5**, **6**, **20**, **27**, and **34** exhibited remarkable antiviral activity against TMV that indicated benzene ring, benzothiadiazole ring, naphthalene ring and phenanthrene ring are suitable for antiviral activity. Among these compouds, *trans*-3-(1,2,3-benzothiadiazole-7-yl)acrylic acid (**20**) and *trans*-3-(2-hydroxyl-1-naphthyl)acrylic acid (**27**) showed the highest antiviral activity which is significantly higher than Ribavirin. The remarkable antiviral activity of *trans*-3-aryl acrylic acids along with their very simple structures give a hope for the future development of new antiviral agents. Further studies on mode of action and toxicity are currently underway in our laboratories.

## Supporting Information

Text S1 **Experimental data of the synthesized compounds.**
(DOC)
