# Supplementary material for: Discovery and SARs of Trans-3-Aryl Acrylic Acids and Their Analogs as Novel Anti- Tobacco Mosaic Virus (TMV) Agents
Source: PLoS One. 2013 Feb 13;8(2):e56475. doi: 10.1371/journal.pone.0056475 (PMC3572066; doi:10.1371/journal.pone.0056475)
Supplement: Text S1 — Experimental data of the synthesized compounds. (DOC) [file pone.0056475.s001.doc]

***Electronic supplementary information (ESI)***

Discovery and SARs of *trans*-3-aryl acrylic acids and their analogs as novel anti- *tobacco mosaic virus* (TMV) agents

*Meng Wu, Ziwen Wang, Chuisong Meng, Kailiang Wang, Yanna Hu, Lizhong Wang,* *and Qingmin Wang**

State Key Laboratory of Elemento-Organic Chemistry, Research Institute of Elemento-Organic Chemistry, Nankai University, Tianjin 300071, China.

Tel: +86-22-23503952; fax: +86-22-23503952; e-mail: [wangqm@nankai.edu.cn](mailto:wangqm@nankai.edu.cn)

**Contents**

Biological assay protocols**………….…………….….……………………………………………………………………………………**S2

Experimental procedures**…………………………………………………………….……………………………………………**S3

References**………………………………………………………………………………….……………………………………………**S8

**Biological assay protocols**

The anti-TMV activity of the synthesized compounds was tested using our previously reported method.[1]

*Antiviral Activity of Compounds against TMV in Vitro.*

Fresh leaf of the 5–6 growth stage of tobacco (*Nicotiana tabacum var Xanthi nc*) inoculated by the juice-leaf rubbing method (concentration of TMV is 5.88 × 10^-2^ *μ*g/mL) was cut into halves along the main vein. The halves were immersed into the solution of 500 µg/mL of the compounds and double distilled water for 20 min, respectively, and then cultured at 25 °C for 72 h. Each compound was replicated at least three times.

*Protective Effect of Compounds against TMV in Vivo.*

The compound solution was smeared on the left side and the solvent serving as control on the right side of growing *Nicotiana tabacum var Xanthi nc* leaves of the same ages. The leaves were then inoculated with the virus after 12 h. A brush was dipped in TMV of 6 × 10-3 mg/mL to inoculate the leaves, which were previously scattered with silicon carbide. The leaves were then washed with water and rubbed softly along the nervature once or twice. The local lesion numbers appearing 3–4 days after inoculation were counted. There are three replicates for each compound.

*Inactivation Effect of Compounds against TMV in Vivo.*

The virus was inhibited by mixing with the compound solution at the same volume for 30 min. The mixture was then inoculated on the left side of the leaves of *Nicotiana tabacum var Xanthi nc*, whereas the right side of the leaves was inoculated with the mixture of solvent and the virus for control. The local lesion numbers were recorded 3–4 days after inoculation. There are three replicates for each compound.

*Curative Effect of Compounds against TMV in Vivo.*

Growing leaves of *Nicotiana tabacum var Xanthi nc* of the same ages were selected. TMV (concentration of 6.0 × 10-3 mg/mL) was dipped and inoculated on the whole leaves. Then the leaves were washed with water and dried. The compound solution was smeared on the left side, and the solvent was smeared on the right side for control. The local lesion numbers were then counted and recorded 3–4 days after inoculation. There are three replicates for each compound.

The *in vitro* and *in vivo* inhibition rates of the compound were then calculated according to the following formula (“av” means average, and controls were not treated with compound).

Inhibition rate (%) = [(av local lesion no. of control − av local lesion no. of drug-treated)/av local lesion no. of control] × 100%

**Experimental procedures**

**Instruments.** 1H NMR spectra were obtained at 400 MHz using a Bruker AC-P 400. Chemical shift values (*δ*) were given in ppm and were downfield from internal tetramethylsilane. High-resolution mass spectra (HRMS) were recorded on FT-ICR MS (Ionspec, 7.0 T). Melting points were determined on an X-4 binocular microscope melting point apparatus (Beijing Tech Instruments Co., Beijing, China) and the thermometer was uncorrected. Reagents were purchased from commercial sources and were used as received. All anhydrous solvent were dried and purified by standard techniques just before use.

**General procedure for the synthesis of *trans-*3-aryl acrylic acids 4**–**11.** A mixture of substituted benzaldehyde (0.065 mol), malonic acid (7.50 g, 0.07 mol), aniline (0.7 mL) and pyridine (8 mL) in toluene (15 mL) was stirred at 85–95 °C for 2 h. Then the mixture was cooled to room temperature and K2CO3 aqueous solution (25%, 35 mL) was added. The mixture was stirred and refluxed again until the solid was dissolved. After cooling, the aqueous phase was separated and acidified with 5% hydrochloric acid aqueous solution. The resulting solid was filtered, washed with water and dried over anhydrous Na2SO4 to give the crude product which was recrystallized from EtOH to give the desired *trans*-3-aryl acrylic acids **4**–**11**. roduct which target compounds.000000000000000000000000000000000000000000000000000000000000000000000000000000000000000000000000

*Data for trans-3-(3,4-dimethoxyphenyl)acrylic acid (****4****)*: yield, 89%; mp 181–183 °C (lit.[2] 179–181 °C); 1H NMR (400 MHz, DMSO-*d6*): δ 12.19 (s, 1H), 7.52 (d, 3*J*HH = 15.6 Hz, 1H), 7.31 (s, 1H), 7.21 (d, 3*J*HH = 8.4 Hz, 1H), 6.98 (d, 3*J*HH = 8.4 Hz, 1H), 6.44 (d, 3*J*HH = 16.0 Hz, 1H), 3.80 (s, 3H), 3.79 (s, 3H).

*Data for trans-3-(3-methoxyphenyl)acrylic acid (****5****)*: yield, 86%; mp 118–120 °C (lit.[3] 119–120 °C); 1H NMR (400 MHz, DMSO-*d6*): δ 12.40 (br, 1H), 7.56 (d, 3*J*HH = 16.0 Hz, 1H), 7.33 (t, 3*J*HH = 8.0 Hz, 1H), 7.24–7.27 (m, 2H), 6.97–6.99 (m, 1H), 6.56 (d, 3*J*HH = 16.0 Hz, 1H).

*Data for trans-3-(3-hydroxyphenyl)acrylic acid (****6****)*: yield, 82%; mp 197–198 °C; 1H NMR (400 MHz, DMSO-*d6*): δ12.37 (s 1H), 9.60 (s, 1H), 7.49 (d, 3*J*HH = 16.0 Hz, 1H), 7.21 (t, 3*J*HH = 8.0 Hz, 1H), 7.10 (d, 3*J*HH = 7.6 Hz, 1H), 7.00 (s, 1H), 6.81–6.84 (m, 1H), 6.40 (d, 3*J*HH = 16.0 Hz, 1H); HRMS (ESI) *m/z* calcd for C9H7O3 [M−H]− 163.0401; found, 163.0405.

*Data for trans-3-(4-hydroxyphenyl)acrylic acid (****7****)*: yield, 73%; mp 213–215 °C (lit.[4] 214 °C); 1H NMR (400 MHz, DMSO-*d6*): δ 12.12 (br, 1H), 9.96 (s, 1H), 7.44–7.55 (m, 3H), 6.79 (d, 3*J*HH = 8.8 Hz, 2H), 6.29 (d, 3*J*HH = 16.0 Hz, 1H).

*Data for trans-3-(4-methoxyphenyl)acrylic acid (****8****)*: yield, 76%; mp 174–175 °C (lit.[5] 172–173 °C); 1H NMR (400 MHz, DMSO-*d6*): δ 12.21 (s, 1H), 7.64 (d, 3*J*HH = 8.8 Hz, 2H), 7.54 (d, 3*J*HH = 16.0 Hz, 1H), 6.97 (d, 3*J*HH = 8.8 Hz, 2H), 6.38 (d, 3*J*HH = 15.6 Hz, 1H), 3.80 (s, 3H).

*Data for trans-3-(3,4,5-trimethoxyphenyl)acrylic acid (****9****)*: yield, 63%; mp 127–129 °C; 1H NMR (400 MHz, DMSO-*d6*): δ 12.30 (br, 1H), 7.53 (d, 2*J*HH = 15.6 Hz, 1H), 7.03 (s, 2H), 6.54 (d, 3*J*HH = 16.0 Hz, 1H), 3.82 (s, 6H), 3.69 (s, 3H); HRMS (ESI) *m/z* calcd for C12H13O6 [M–H]–­ 237.0768; found 237.0767.

*Data for trans-3-(3,4-methylenedioxyphenyl)acrylic acid (****10****)*: yield, 78%; mp 212–213 °C; 1H NMR (400 MHz, DMSO-*d6*): δ 12.25 (s, 1H), 7.51 (d, 3*J*HH = 16.0 Hz, 1H), 7.37 (d, 4*J*HH = 1.2 Hz, 1H), 7.12–7.17 (m, 1H), 6.95 (d, 3*J*HH = 8.0 Hz, 1H), 6.40 (d, 3*J*HH = 16.0 Hz, 1H), 6.08 (s, 2H); HRMS (ESI) *m/z* calcd for C10H7O4 [M–H]–­ 191.0350; found 191.0353.

*Data for trans-3-(3,4-ethylenedioxyphenyl)acrylic acid (****11****)*: yield, 83%; mp 186–188 °C; 1H NMR (400 MHz, DMSO-*d6*): δ 12.24 (s, 1H), 7.48 (d, 3*J*HH = 16.0 Hz, 1H), 7.23 (d, 4*J*HH = 1.6 Hz, 1H), 7.16–7.18 (m, 1H), 6.88 (d, 3*J*HH = 8.4 Hz, 1H), 6.37 (d, 3*J*HH = 16.0 Hz, 1H), 4.27 (d, 3*J*HH = 3.2 Hz, 4H); HRMS (ESI) *m/z* calcd for C11H9O4 [M–H]- 205.0506; found, 205.0508.

**Synthesis of 3-(4-hydroxy-3-methoxyphenyl)propanoic acid (28)**. The stirred mixture of *trans*-ferulic acid (**1**) (1.94 g, 0.01 mol) and 10% Pd/C (0.3 g) in ethanol (300 mL) was bubbled H2 for 10 h at room temperature, and then filtered and concentrated in vacuo to give **12** as a light yellow solid (1.90 g, 97%), mp 81–83 °C (lit.[6] 86–87 °C); 1H NMR (400 MHz, DMSO-*d6*): δ 6.78 (s, 1H), 6.66 (d, 3*J*HH = 8.0 Hz, 1H), 6.59 (d, 3*J*HH = 8.0 Hz, 1H), 3.74 (s, 3H), 2.71 (t, 3*J*HH = 7.6 Hz, 2H), 2.47 (t, 3*J*HH = 7.6 Hz, 2H).

**Synthesis of 6-benzyloxy-2,3-dimethoxyphenanthren-9-carboxaldehyde (36m)**. To a solution of (6-benzyloxy-2,3-dimethoxyphenanthren-9-yl)methanol (**37**)(2.00 g, 5.35 mmol) in dichloromethane (100 mL) was added PCC (2.31 g, 10.70 mmol) at room temperature. The mixture was stirred for 3 h and then filtered, evaporated to give the crude product. The crude was purified by column chromatography (petroleum ether/EtOAc, 4:1, v/v) to give **36m** (1.95 g, 98%) as a white solid, mp 200–201 ºC; 1H NMR (400 MHz, CDCl­3): δ 10.26 (s, 1 H), 9.33 (d, 3*J*HH = 9.2 Hz, 1 H), 8.00 (s, 1 H), 7.94 (d, 4*J*HH = 2.0 Hz, 1 H), 7.77 (s, 1 H), 7.54 (d, 3*J*HH = 7.6 Hz, 2 H),7.38–7.43 (m, 4 H), 7.31 (s, 1 H), 5.29 (s, 2 H), 4.12 (s, 3 H), 4.06 (s, 3 H). HRMS (ESI): calcd. for C24H21O4 [M+H]+ 373.1434; found 373.1437.

**Synthesis of 6-hydroxyl-2,3-dimethoxy-phenanthren-9-carboxaldehyde (36n)**.To a solution of 6-hydroxy-2,3-dimethoxyphenanthrene-9-carboxylic acid methyl ester (**38**) (2.24 g, 7.18 mmol) in DMF (20 mL) was added imidazole (2.46 g, 35.90 mmol) and *tert*-butyldimethylsilyl chloride (2.70 g, 17.94 mmol). The mixture was stirred for 3 h at room temperature and then ammonium chloride solution was added. The solution was extracted with ethyl acetate and then the combined organic phase was dried over anhydrous Na2SO4, filtered and evaporated. The crude was purified by column chromatography (petroleum ether/EtOAc, 5:1, v/v) to give **40** (2.76 g, 90%) as a white solid. To the solution of **40** (2.42 g, 5.67 mmol) in THF (60 mL) was added LiAlH4 (0.43 g, 11.35 mmol) at 0 ºC. The mixture was stirred for 1.5 h at room temperature and then cooled to 0 ºC, dichloromethane (100 mL) was added and then water (70 mL) was added slowly. The organic layer was separated, and the water phase was extracted with dichloromethane. The combined organic phase was dried over anhydrous Na2SO4, filtered and evaporated to give a white solid. The solid was redissolved in dichloromethane (60 mL), and PCC (2.45 g, 11.35 mmol) was added. The solution was stirred for 3 h and then filtered and evaporated. The residue was dissolved in THF (60 mL), and tetrabutyl ammonium fluoride (2.96 g, 11.35 mmol) was added. After the solution was stirred for 1 h, dichloromethane (100 mL) and water (100 mL) was added. The organic layer was separated, and the water phase was extracted with dichloromethane. The combined organic phase was dried over anhydrous Na2SO4, filtered and evaporated to give crude product. The crude product was purified by column chromatography (petroleum ether/EtOAc, 2:1, v/v) to give **36n** **(**1.95 g, 73%) as a yellow solid.

*Data for 6-tert-butyldimethylsilyloxy-2,3-dimethoxyphenanthrene-9-carboxylic acid methyl ester(****40****)*: mp 105–106 ºC; 1H NMR (400 MHz, CDCl­3): δ 8.87 (d, 3*J*HH = 9.2 Hz, 1 H), 8.31 (s, 1 H), 7.91 (d, 3*J*HH = 2.4 Hz, 1 H), 7.84 (s, 1 H), 7.27 (s, 1 H), 7.19 (dd, 3*J*HH = 9.2 Hz, 4*J*HH = 2.4 Hz, 1 H), 4.14 (s, 3 H), 4.04 (s, 3 H), 4.01 (s, 3 H), 1.06 (s, 9 H), 0.31 (s, 6 H). HRMS (ESI): calcd. for C24H31O5Si [M+H]+ 427.1935; found 427.1942.

*Data for 6-hydroxyl-2,3-dimethoxy-phenanthren-9-carboxaldehyde (****36n****)*: mp 217–219 °C; 1H NMR (400 MHz, DMSO-*d6*): δ 10.18 (s, 1 H), 9.99 (s, 1 H), 9.12 (d, 3*J*HH = 8.4 Hz, 1 H), 8.15 (s, 1 H), 8.03 (s, 1 H), 7.92 (s, 1 H), 7.53 (s, 1 H), 7.24 (d, 3*J*HH = 8.4 Hz, 1 H), 4.06 (s, 3 H), 3.94 (s, 3 H).HRMS (ESI): calcd. for C17H15O4 [M+H]+ 283.0965; found 283.0964.

**Synthesis of 3-hydroxyl-6,7-dimethoxy-phenanthren-9-carboxaldehyde (36o).** A procedure analogous to the preparation of **36n** was used. 3-Hydroxy-6,7-dimethoxyphenanthrene-9-carboxylic acid methyl ester (**39**) gave **41** as a white solid and **36o** as a yellow solid.

*Data for 3-tert-butyldimethylsilyloxy-6,7-dimethoxyphenanthrene-9-carboxylic acid methyl ester(****41****)*: yield, 94%; mp 95–96 °C; 1H NMR (400 MHz, CDCl­3): δ 8.65 (s, 1 H), 8.45 (s, 1 H), 7.85–7.87 (m, 2 H), 7.83 (d, 3*J*HH = 8.4 Hz, 1 H), 7.13 (dd, 3*J*HH = 8.4 Hz, 4*J*HH = 2.4 Hz, 1 H), 4.13 (s, 3 H), 4.08 (s, 3 H), 4.02 (s, 3 H), 1.06 (s, 9 H), 0.32 (s, 6 H). HRMS (ESI): calcd. for C24H31O5Si [M+H]+ 427.1935; found 427.1942.

*Data for 6-hydroxyl-2,3-dimethoxy-phenanthren-9-carboxaldehyde (****36o****)*: yield, 70%; mp 229–230 °C; 1H NMR (400 MHz, DMSO-*d6*): δ 10.41 (s, 1 H), 10.21 (s, 1 H), 8.83 (s, 1 H), 8.35 (s, 1 H),8.01–8.03 (m, 2 H), 7.99 (s, 1 H), 7.22 (dd, 3*J*HH = 8.4 Hz, 4*J*HH = 2.0 Hz, 1 H), 4.03 (s, 3 H), 3.93 (s, 3 H). HRMS (ESI): calcd. for HRMS (ESI): calcd. for C17H15O4 [M+H]+ 283.0965; found 283.0967.

**General procedure for the synthesis of *trans-*3-aryl acrylic acids 12**–**26.** A mixture of aromatic aldehyde (**36a**–**o**) (2.16 mol), malonic acid (0.45 g, 4.32 mol), aniline (2.0 mL) and pyridine (20 mL) were stirred at reflux for 8–10 h. The reaction mixture was cooled to room temperature and 25 mL of 20% K2CO3 (aq.) was added. The mixture was stirred and refluxed and then cooled to room temperature. Dichloromethane (20 mL) was added and then the aqueous phase was separated and acidified with 5% hydrochloric acid aqueous solution. The resulted solid was filtered, washed with water and dried anhydrous Na2SO4 to give the crude product which was purified by recrystallization or column chromatography to give the desired *trans*-3-aryl acrylic acids **12**–**26**.

*Data for* *trans*-*3-(2-Furyl)acrylic acid(****12****):* Through column chromatography (petroleum ether/EtOAc, 2:1, v/v) to give **12** as a yellow solid. Yield, 66%; mp 143–144 °C (lit.[7] 140–142 °C); 1H NMR (400 MHz, CDCl­3): δ7.53 (d, 3*J*HH = 15.6 Hz, 1 H), 7.52 (d, 3*J*HH = 1.6 Hz, 1 H), 6.68 (d, 3*J*HH = 3.6 Hz, 1 H), 6.50 (dd, 3*J*HH = 3.6 Hz, 3*J*HH = 1.6 Hz, 1 H), 6.32 (d, 3*J*HH = 15.6 Hz, 1 H).

*Data for trans-3-(2-thienyl)acrylic acid (****13****)*: Through column chromatography (petroleum ether/EtOAc, 2:1, v/v) to give **13** as a yellow solid. Yield, 69%; mp 145–148 °C (lit.[8] 144–146 °C); 1H NMR (400 MHz, CDCl­3): δ7.89 (d, 3*J*HH = 15.6 Hz, 1 H), 7.52 (d, 3*J*HH = 5.2 Hz, 1 H), 7.31 (d, 3*J*HH = 3.2 Hz, 1 H), 7.08 (dd, 3*J*HH = 5.2 Hz, 3*J*HH = 3.2 Hz, 1 H), 6.25 (d, 3*J*HH = 15.6 Hz, 1 H).

*Data for trans-3-(2-pyridyl)acrylic acid (****14****)*: The crude solid was washed with ether to give **14** as a yellow solid. Yield, 65%; mp 199–201 °C; 1H NMR (400 MHz, DMSO-*d6*): δ12.63 (s, 1 H), 8.64 (d, 3*J*HH = 4.0 Hz, 1 H), 7.52 (td, 3*J*HH = 7.6 Hz, 4*J*HH = 1.6 Hz, 1 H), 7.60 (d, 3*J*HH = 15.6 Hz, 1 H), 7.08 (dd, 3*J*HH = 7.2 Hz, 3*J*HH = 4.8 Hz, 1 H), 6.83 (d, 3*J*HH = 15.6 Hz, 1 H). HRMS (ESI): *m/z* calcd. for C8H8NO2 [M+H]+ 150.0550; found 150.0550.

*Data for trans-3-(3-pyridyl)acrylic acid (****15****)*: The crude solid was washed with ether to give **15** as a yellow solid. Yield, 82%; mp 236–238 °C (lit.[9] 232–234 °C); 1H NMR (400 MHz, DMSO-*d6*): δ 8.86 (s, 1 H), 8.59 (d, 3*J*HH = 4.4 Hz, 1 H), 8.16 (d, 3*J*HH = 7.6 Hz, 1 H), 7.62 (d, 3*J*HH = 8.4 Hz, 1 H), 7.44–7.47 (m, 1 H), 6.71 (d, 3*J*HH = 8.4 Hz, 1 H).

*Data for trans-3-(4-pyridyl)acrylic acid (****16****)*: The crude solid was washed with ether to give **16** as a yellow solid. Yield, 87%; mp 277–279 °C (lit.[10] 278–280 °C); 1H NMR (400 MHz, DMSO-*d6*): δ 12.78 (s, 1 H), 8.62 (d, 3*J*HH = 6.0 Hz, 2 H), 7.66 (d, 3*J*HH = 6.0 Hz, 2 H), 7.56 (d, 3*J*HH = 16.0 Hz, 1 H), 6.80 (d, 3*J*HH = 16.0 Hz, 1 H).

*Data for trans-3-(2-thiazolyl)acrylic acid (****17****)*: Through column chromatography (petroleum ether/EtOAc, 2:1, v/v) to give **17** as a white solid. Yield, 80%; mp 186–189 °C; 1H NMR (400 MHz, DMSO-*d6*): δ 12.81 (s, 1 H), 8.01 (d, 3*J*HH = 3.2 Hz, 1 H), 7.94 (d, 3*J*HH = 3.2 Hz, 1 H), 7.70 (d, 3*J*HH = 15.6 Hz, 1 H), 6.67 (d, 3*J*HH = 15.6 Hz, 1 H). HRMS (ESI): *m/z* calcd. for C6H6NO2S [M+H]+ 156.0114; found 156.0112.

*Data for trans-3-(5-methyl-3-isoxazolyl)acrylic acid (****18****)*: Through column chromatography (petroleum ether/EtOAc, 2:1, v/v) to give **18** as a yellow solid. Yield, 64%; mp 184–185 °C (lit.[11] 175–177 °C); 1H NMR (400 MHz, DMSO-*d6*): δ 12.89 (s, 1 H), 7.41 (d, 3*J*HH = 16.0 Hz, 1 H), 6.77 (s, 1 H), 6.69 (d, 3*J*HH = 15.6 Hz, 1 H), 2.44 (s, 3 H).

*Data for trans-3-(5-chlorine-3-methyl-1-phenyl-4-pyrazolyl)acrylic acid (****19****)*: The crude solid was recrystallized from ethyl acetate to give **19** as a white solid. Yield, 64%; mp 202–203 °C; 1H NMR (400 MHz, CDCl­3): δ 7.73 (d, 3*J*HH = 16.0 Hz, 1 H), 7.45–7.56 (m, 5 H), 6.42 (d, 3*J*HH = 16.0 Hz, 1 H), 2.48 (s, 3 H). HRMS (ESI): *m/z* calcd. for C13H12ClN2O2 [M+H]+ 263.0582; found 263.0579.

*Data for trans-3-(1,2,3-benzothiadiazole-7-yl)acrylic acid (****20****)*: The crude solid was washed with ether to give **20** as a white solid. Yield, 64%; mp 212–214 °C; 1H NMR (400 MHz, DMSO-*d6*): δ 12.87 (s, 1 H), 8.85 (d, 3*J*HH = 8.0 Hz, 1 H), 8.85 (d, 3*J*HH = 7.2 Hz, 1 H), 7.95 (d, 3*J*HH = 16.0 Hz, 1 H), 7.89 (t, 3*J*HH = 7.6 Hz, 1 H), 6.46 (d, 3*J*HH = 16.0 Hz, 1 H). HRMS (ESI): *m/z* calcd. for C9H7N2O2S [M + H]+ 207.0223; found 207.0220.

*Data for trans-3-(6-hydroxyl-2-naphthyl)acrylic acid (****21****)*: The crude solid was washed with ether to give **21** as a white solid. Yield, 73%; mp 238–240 °C; 1H NMR (400 MHz, DMSO-*d6*): δ 8.03 (s, 1 H), 7.80 (d, 3*J*HH = 8.8 Hz, 1 H), 7.75 (dd, 3*J*HH = 8.8 Hz, 4*J*HH = 1.6 Hz, 1 H), 7.66–7.71 (m, 2 H), 7.10–7.14 (m, 2 H), 6.55 (d, 3*J*HH = 16.0 Hz, 1 H). HRMS (ESI): *m/z* calcd. for C13H11O3 [M+H]+ 215.0703; found 215.0700.

*Data for trans-3-(2,3,6,7-tetramethoxyphenanthren-9-yl)acrylic acid (****22****)*: The crude solid was recrystallized from ethanol to give **22** as a yellow solid. Yield, 70%; mp 269–271 °C; 1H NMR (400 MHz, DMSO-*d6*): δ 12.54 (s, 1 H), 8.36 (d, 3*J*HH = 15.6 Hz, 1 H), 8.08 (s, 1 H), 8.06 (s, 1 H), 7.99 (s, 1 H), 7.47 (s, 1 H), 7.46 (s, 1 H), 6.60 (d, 3*J*HH = 15.6 Hz, 1 H), 4.05 (s, 6 H), 3.97 (s, 3 H), 3.92 (s, 3 H). HRMS (ESI): *m/z* calcd. for C21H21O6 [M+H]+ 369.1333; found 339.1336.

*Data for trans-3-(3,6,7-trimethoxyphenanthren-9-yl)acrylic acid (****23****)*: The crude solid was recrystallized from ethanol to give **23** as a yellow solid. Yield, 77%; mp 277–280 °C; 1H NMR (400 MHz, DMSO-*d6*): δ 12.52 (s, 1 H), 8.36 (d, 3*J*HH = 15.6 Hz, 1 H), 8.15 (s, 1 H), 8.13 (s, 1 H), 8.08 (d, 3*J*HH = 2.0 Hz, 1 H), 7.94 (d, 3*J*HH = 8.8 Hz, 1 H), 7.49 (s, 1 H), 7.26 (dd, 3*J*HH = 8.8 Hz, 4*J*HH = 2.0 Hz, 1 H), 6.63 (d, 3*J*HH = 15.6 Hz, 1 H), 4.06 (s, 3 H), 4.02 (s, 3 H), 3.99 (s, 3 H). HRMS (ESI): *m/z* calcd. for C20H19O5 [M+H]+ 339.1227; found 339.1227.

*Data for trans-3-(6-benzyloxy-2,3-dimethoxyphenanthren-9-yl)acrylic acid (****24****)*: The crude solid was recrystallized from ethanol to give **24** as a yellow solid. Yield, 76%; mp 196–197 °C; 1H NMR (400 MHz, DMSO-*d6*): δ 12.52 (s, 1 H), 8.35 (d, 3*J*HH = 15.6 Hz, 1 H), 8.25 (d, 3*J*HH = 2.4 Hz, 1 H), 8.12 (d, 3*J*HH = 9.2 Hz, 1 H), 8.08 (s, 1 H), 8.05 (s, 1 H), 7.59 (d, 3*J*HH = 7.2 Hz, 2 H), 7.51 (s, 1 H), 7.44 (t, 3*J*HH = 7.2 Hz, 2 H), 7.34–7.40 (m, 2 H), 6.59 (d, 3*J*HH = 15.6 Hz, 1 H), 5.39 (s, 2 H), 4.06 (s, 3 H), 3.93 (s, 3 H). HRMS (ESI): *m/z* calcd. for C26H23O5 [M+H]+ 415.1540; found 415.1543.

*Data for trans-3-(2,3-dimethoxy-6-hydroxyl-phenanthren-9-yl)acrylic acid (****25****)*: Through column chromatography (Dichloromethane /Methanol, 25:1, v/v) to give **25** as a yellow solid. Yield, 68%; mp 239–241 °C; 1H NMR (400 MHz, DMSO-*d6*): δ 12.51 (s, 1 H), 9.89 (s, 1 H), 8.34 (d, 3*J*HH = 15.6 Hz, 1 H), 8.04 (d, 3*J*HH = 9.6 Hz, 2 H), 8.00 (s, 1 H), 7.92 (s, 1 H), 7.48 (s, 1 H), 7.19 (d, 3*J*HH = 8.8 Hz, 1 H), 6.57 (d, 3*J*HH = 15.6 Hz, 1 H), 4.03 (s, 3 H), 3.92 (s, 3 H). HRMS (ESI): *m/z* calcd. for C19H17O5 [M+H]+ 325.1071; found 325.1072.

*Data for trans-3-(6,7-dimethoxy-3-hydroxyl-phenanthren-9-yl)acrylic acid (****26****)*: Through column chromatography (Dichloromethane /Methanol, 25:1, v/v) to give **26** as a yellow solid. Yield, 68%; mp 239–241 °C; 1H NMR (400 MHz, DMSO-*d6*): δ 12.48 (s, 1 H), 9.99 (s, 1 H), 8.34 (d, 3*J*HH = 15.6 Hz, 1 H), 8.08 (s, 1 H), 7.98 (s, 1 H), 7.95 (d, 3*J*HH = 1.6 Hz, 2 H), 7.86 (d, 3*J*HH = 8.8 Hz, 1 H), 7.47 (s, 1 H), 7.13 (dd, 3*J*HH = 8.8 Hz, 3*J*HH = 2.0 Hz, 1 H), 6.60 (d, 3*J*HH = 15.6 Hz, 1 H), 4.02 (s, 3 H), 3.98 (s, 3 H). HRMS (ESI): *m/z* calcd. for C19H17O5 [M+H]+ 325.1071; found 325.1075.

**Synthesis of *trans*-3-(2-hydroxyl-1-naphthyl)acrylic acid (27)**. 2-Hydroxy-1-naphthaldehyde (**36p**, 1.50 g, 8.72 mmol) and (carbethoxymethyl)triphenylphosphonium bromide (3.73 g, 8.72 mmol) was dissolved in methanol (20 mL). Then the solution was slowly added to a solution of potassium carbonate (2 g) in methanol (20 mL). The mixture was stirred for 6 h at room temperature and evaporated to remove the methanol. Dichloromethane and water were added and the he aqueous phase was extracted with dichloromethane. The combined organic phase was dried over anhydrous Na2SO4, filtered and evaporated to give a white solid. The solid was added to a solution of 2 mol/L aqueous potassium hydroxide (30 mL) and stirred for 2 h. Dichloromethane (50 mL) was added and then the aqueous phase was separated and acidified with 2 mol/L hydrochloric acid aqueous solution. The resulted solid was filtered, washed with water and dried to give the crude product which was recrystallized from ethyl acetate to give 1.34 g of **27** as a white solid, yield, 72%; mp 179–180°C (lit.[12] 165 °C dec); 1H NMR (400 MHz, DMSO-*d6*): δ 12.32 (s, 1 H), 10.75 (s, 1 H), 8.24 (d, 3*J*HH = 16.0 Hz, 1 H), 8.10 (d, 3*J*HH = 8.0 Hz, 1 H), 7.85 (d, 3*J*HH = 8.0 Hz, 2 H), 7.55 (br, 1 H), 7.36 (br, 1 H), 7.28 (d, 3*J*HH = 8.4 Hz, 1 H), 6.82 (d, 3*J*HH = 16.0 Hz, 1 H).

**General procedure for the synthesis of *trans-*3-aryl methylacrylate 30-34**.To a solution of*trans*-3-Aryl acrylic acid **1**, **5**, **6**, **20** or **27** (2 mmol) in dry methanol (25 mL) was added concentrated sulfuric acid (0.72 g). The solution was stirred at reflux for 8 h. Most of the methanol was removed by rotary evaporation, the residue was redissolved in dichloromethane and washed with saturated sodium hydrogen carbonate. The organic phase was dried over anhydrous Na2SO4, filtered and evaporated to give the products.

*Data for methyl trans-3-(4-hydroxy-3-methoxyphenyl)acrylate (****30****)*: a white solid; yield, 95%; mp 48–49 °C (lit.[13] 49–51 °C); 1H NMR (400 MHz, CDCl3) δ 9.61 (s, 1H), 7.56 (d, 3*J*HH = 16.0 Hz, 1H), 7.32 (d, 4*J*HH = 1.6 Hz, 1H), 7.09–7.16 (m, 1H), 6.80 (d, 3*J*HH = 8.0 Hz, 1H), 6.48 (d, 3*J*HH = 15.6 Hz, 1H), 3.82 (s, 3H), 3.70 (s, 3H).

*Data for methyl trans-3-(3-methoxyphenyl)acrylate (****31****)*: a white oil, yield, 97%; 1H NMR (400 MHz, CDCl3) δ 7.66 (d, 3*J*HH = 16.0 Hz, 1H), 7.30 (t, 3*J*HH = 8.0 Hz, 1H), 7.11 (d, 3*J*HH= 7.6 Hz, 1H), 7.04 (s, 1H), 6.93 (dd, 3*J*HH = 8.0, 4*J*HH = 2.4 Hz, 1H), 6.43 (d, 3*J*HH = 16.0 Hz, 1H), 3.82 (s, 3H), 3.80 (s, 3H).

*Data for methyl trans-3-(3-hydroxyphenyl)acrylate (****32****)*: a white solid; yield, 98%; mp 88–90 °C; 1H NMR (400 MHz, CDCl3) δ 7.65 (d, 3*J*HH = 16.0 Hz, 1H), 7.25–7.28 (m, 1H), 7.10 (d, 3*J*HH = 7.6 Hz, 1H), 7.03 (br, 1H), 6.89 (dd, 3*J*HH = 8.0, 4*J*HH = 2.0 Hz, 1H), 6.42 (d, 3*J*HH = 16.0 Hz, 1H), 3.82 (s, 3H).

*Data for methyl trans-3-(1,2,3-benzothiadiazole-7-yl)acrylate(****33****)*: a white solid; yield, 94%; mp 118–120 °C; 1H NMR (400 MHz, CDCl3): δ8.70 (d, 3*J*HH = 8.4 Hz, 1 H), 7.95 (d, 3*J*HH = 16.0 Hz, 1 H), 7.84 (d, 3*J*HH = 7.2 Hz, 1 H), 7.73 (t, 3*J*HH = 8.0 Hz, 1 H), 6.46 (d, 3*J*HH = 16.0 Hz, 1 H), 3.88 (s, 3 H).

*Data for methyl trans-3-(2-hydroxyl-1-naphthyl)acrylate(****34****)*: a white solid; yield, 96%; mp 166–168 °C; 1H NMR (400 MHz, CDCl3): δ8.33 (d, 3*J*HH = 16.4 Hz, 1 H), 8.02 (d, 3*J*HH = 8.8 Hz, 1 H), 7.76–7.79 (m, 2 H), 7.53 (t, 3*J*HH = 7.6 Hz, 1 H), 7.39 (t, 3*J*HH = 7.6 Hz, 1 H), 7.14 (d, 3*J*HH = 8.8 Hz, 1 H), 6.76 (d, 3*J*HH = 16.4 Hz, 1 H), 6.07 (s, 1 H), 3.89 (s, 1 H). HRMS (ESI): calcd. for C14H13O3 [M–H]–­ 227.0714; found 227.0710.

**References**

1. Wang KL, Su B, Wang ZW, Wu M, Li Z, et al. (2010) Synthesis and antiviral activities of phenanthroindolizidine alkaloids and their derivatives. *J. Agric. Food Chem.* 58: 2703–2709.
2. Li X, Wang Y, Wu J, Li Y, Wang Q, et al. (2009) Novel aminopeptidase N inhibitors derived from antineoplaston AS2–5 (Part II). *Bioorg. Med. Chem.* 17: 3061–3071.
3. Cleland GH (1969) The meerwein reaction in amino acid synthesis. 11. An investigation of twenty-one substituted anilines. *J. Org. Chem.* 34: 744–747.
4. Mogilaiah K, Reddy GR (2004) Microwave-assisted solvent-free synthesis of trans-cinnamic acids using lithium chloride as catalyst. *Synth. Commun.* 34: 205–210.
5. Yuan D, Zhang Q (2008) Heck reaction catalyzed by polystyrene supported dicyclohexylamine palladium. *Industrial Catalysis* 16: 62–65.
6. Takahashi T, Miyazawa M (2010) Tyrosinase inhibitory activities of cinnamic acid analogues. *Pharmazie* 65: 913–918.
7. Niels E (1956) Transformation of 2-(β,β-dicarbomethoxyethyl)furan into m-hydroxybenzoic acid. *Acta. Chem. Scan.* 10: 1664–1666.
8. Zhu M, Song YL, Cao Y (2007) A fast and convenient Heck reaction in water under microwave irradiation*. Synthesis* 6: 853–856.
9. Bellassoued M, Lensen N, Bakasse M, Mouelhi S (1998) Two-Carbon homologation of aldehydes via silyl ketene acetals: a new stereoselective approach to (*E*)-alkenoic acids. *J. Org. Chem.* 63: 8785–8789.
10. Tetsuhashi M, Ishikawa M, Hashimoto M, Hashimoto Y, Aoyama H (2010) Development of tryptase inhibitors derived from thalidomide. *Bioorg. Med. Chem.* 18: 5323–5338.
11. Kuo CJ, Shie JJ, Fang JM, Yen GR, Hsu JT, et al. (2008) Design, synthesis, and evaluation of 3C protease inhibitors as anti-enterovirus 71 agents. *Bioorg. Med. Chem.* 16: 7388–7398.
12. Dey BB, Rao RHR, Sankaranarayanan (1932) Stability of coumaric acids derived from beta.alpha.-1,2-naphthopyrones. *J. Indian Che. Soc.*9: 281–289.
13. Bisogno F, Mascoti L, Sanchez C, Garibotto F, Giannini F, et al. (2007) Structure-antifungal activity relationship of cinnamic acid derivatives. *J. Agric. Food Chem.* 55: 10635–10640.
